# Improving of psychological status and inflammatory biomarkers during omalizumab for chronic spontaneous urticaria

**DOI:** 10.2144/fsoa-2020-0087

**Published:** 2020-08-10

**Authors:** Laura Diluvio, Arianna Piccolo, Francesco Marasco, Laura Vollono, Caterina Lanna, Barbara Chiaramonte, Cinzia Niolu, Elena Campione, Luca Bianchi

**Affiliations:** 1 Dermatology, Department of Systems Medicine, University of Rome Tor Vergata, Viale Oxford, Rome 81 00133, Italy; 2 Psychiatry, Department of Systems Medicine, University of Rome Tor Vergata, Viale Oxford, Rome 81 00133, Italy; 3 INAIL, Actuarial-Statistic consultancy office, via Stefano Gradi, Rome 55 00143, Italy

**Keywords:** anxiety, chronic spontaneous urticaria, CSU, depression, DLQI, H1-anti-histamine, HADS, Hospital Anxiety and Depression Scale, omalizumab, psychiatric disorders, UAS7

## Abstract

**Background:**

Depression and anxiety are the most common psychiatric comorbidities in chronic spontaneous urticaria (CSU). Omalizumab is a monoclonal antibody approved for CSU treatment. We evaluated the prevalence of anxiety and depression in CSU patients before and after treatment with omalizumab.

**Materials & methods:**

A total of 30 patients were enrolled in the study: 15 patients affected by CSU and treated with omalizumab and the other 15 healthy subjects did not receive any systemic therapy. All patients were evaluated using Hospital Anxiety and Depression Scale, CRP and erythrocyte sedimentation rate, at baseline and after 6 months.

**Results:**

The omalizumab group after 6 months of therapy had a decrease of all the scores and biomarkers.

**Conclusion:**

Omalizumab allowed an improvement of urticaria and mental comorbidities.

## Lay abstract

Depression and anxiety are the most common psychiatric comorbidities in chronic spontaneous urticaria (CSU). We evaluated the prevalence of anxiety and depression in CSU cases and serum levels of inflammatory biomarkers in CSU patients affected also by psychiatric disorders before and after treatment with omalizumab. Omalizumab induced an improvement of urticaria and concurrent mental comorbidities.

Chronic spontaneous urticaria (CSU) refers to a disturbing and detrimental disease characterized by the presence of daily itchy wheals over 6 weeks, with or without angioedema, in the absence of a specific triggering cause. CSU represents the most frequent type of urticaria and occurs in approximately 1% of the general population, especially women between 20 and 40 years old [1]. The prevalence of CSU in Italy is reported to be approximately 0.38%, with an annual incidence of 1.50/1000 person-years [2]. Establishing the causes of urticaria remains an unnerving challenge, leading to frustration and a reduction in the patient’s quality of life (QoL) [3]. The occurrence of angioedema has been associated with a longer disease duration compared with patients with skin disorder alone [4]. Moreover, angioedema episodes may be disfiguring or painful, limiting daily activities and social interactions, and may have a significant impact on the QoL of CSU patients [5]. Nonsedating H1-antihistamines represent the treatment of choice for CSU, though add-on therapies may be necessary for the control of signs and symptoms in approximately half of patients [6]. Omalizumab is an IgG1k monoclonal antibody that blocks the binding of free IgE to its receptor (FcεRI) on the surface of effector cells and was approved by US FDA (FDA, MD, USA) for the treatment of CSU in 2014. In addition to the remission of symptoms, urticaria therapy should also aim to improve the QoL, which appears to be poor in these patients [3]. Depression and anxiety are the most common psychiatric comorbidities identified in CSU patients and these psychiatric disorders, may in turn have an impact on QoL [7,8]. The mechanisms leading to poor psychological status in CSU patients are not completely understood and whether the presence of depression and anxiety prior to the onset of CSU can worsen symptoms remains unclear.

In this study, we compiled our experience regarding the use of omalizumab in patients with urticaria and concurrent mental comorbidities, such as anxiety and depression. Psychiatric examinations of all patients and control groups were performed according to the diagnostic and statistical manual of mental disorders (DSM-IV) criteria [9]. Hospital Anxiety and Depression Scale (HADS) was applied to those who were found affected by anxiety or depression [10]. A study involving patients with coronary heart disease confirmed that HADS is a reliable and valid screening instrument for anxiety and depression [11]. In addition, we decided to evaluate systemic inflammation changes in serum markers, such as CRP and ESR during the use of omalizumab. Elevated levels of CRP, an acute-phase protein that serves as an early and sensitive marker of inflammation, have been consistently reported in CSU patients and its assessment may be helpful in the management of this condition [12]. Besides CRP, serum ESR should be considered in patients with severe urticaria refractory to treatment [13]. CRP and ESR, considered as independent risk factors for coronary artery disease and markers for systemic inflammation, are reported to be increased in depression [3,14]. Depression is associated with immunological changes, thus it is possible that depression in turn may affect the development of coronary disease through systemic inflammation [15]. Similar to depression, anxiety disorder was associated with elevated levels of CRP and ESR, even after elimination of confounding factors. It has been hypothesized that a low-grade inflammation may play a role in the etiology of anxiety disorders and may represent a link between anxiety disorders and cardiovascular burden. Anxiety disorders are also highly comorbid with depression, which has been associated with immune dysregulation [16,17]. The aim of the present study is to examine the prevalence of accompanying anxiety and depression in CSU cases and serum levels of inflammatory biomarkers, as CSR and ESR in CSU patients affected also by psychiatric disorders before and after treatment with omalizumab.

## Patients & methods

### Demographic & clinic data

The study included a total of 30 patients over 18 years of age (age 23 ± 35 year; 10 M/20F). A total of 15 individuals were affected by moderate-to-severe CSU (defined as Urticaria Activity Score for 7 days [UAS7] >28) and treated with monoclonal antibody omalizumab. Another 15 subjects were enrolled as the control group. These subjects did not receive systemic therapy. There was no significant demographic difference in age (p = 0.24) and gender (p = 0.19) between the patient group and the control group. Data collection lasted 12 months, from January to December 2019, at the Urticaria Clinic of the Dermatology Department of Policlinico Tor Vergata, (Rome, Italy). The study was approved by the local Ethics Committee in accordance with the ethical principles of the Declaration of Helsinki and was consistent with the guidelines for good clinical practice. Written informed consent was obtained from all patients included in the study. Patients were excluded if they had other chronic dermatological diseases, another immune inflammatory condition, a history of long-term psychiatric disorders, use of psychotropic substances within 3 months, marked cognitive impairment or were unable to give consent.

### Laboratory & dermatological assessments

All patients underwent complete blood count, liver and renal function tests, electrophoresis, fibrinogen, parasitology stool test, thyroid function test, antithyroid antibody test, antinuclear antibody test, extractable nuclear antigen test, IgE level assessment paper radioimmuno sorbent test (PRIST), CRP and ESR. Clinical assignment of UAS7 and Dermatology Life Quality Index (DLQI) at baseline, 12 weeks (W12), 24 weeks (W24) and 52 weeks (W52) of treatment was performed for all patients [18]. ESR was detected by classic Westergren method (MicroSED-10^®^ System, Electa Lab, Italy); turbidimetric inhibition (immunoassay QuikRead^®^101 CRP analyzer, Orion Diagnostica Oy, Espoo, Finland) was applied to investigate CRP levels in blood specimens. All testing progress was executed as indicated in the manufacturers’ instructions. The normal range for ESR was 0–15 mm/h (male) or 0–20 mm/h (female) and was 0–8 mg/l for CRP. UAS7, which ranges from 0 to 42, was performed to assess the disease activity in CSU patients. UAS7, disease duration, medications, age, gender, marital status, comorbidities, occupation and income were recorded. DLQI is an assessment scale including ten questions concerning patients’ perception of the impact of cutaneous conditions on different aspects of their health-related QoL over the last week. Four possible responses to each item are: ‘very much’, ‘a lot’, ‘a little’, ‘not at all’. Patients treated with omalizumab received 300 mg subcutaneous injection as add-on to H1-antihistamines administered every 4 weeks for 6 months, followed by an 8-week treatment interruption. In case of recurrence, a second cycle of five additional doses of omalizumab 300 mg every 4 weeks (5 months) was administered. Clinical response was assessed based on reduction of UAS7 (outcome were classified as complete response [≤6]) and DLQI (outcomes were classified as complete [<6], partial [6–10] or no response [>10]). Safety was monitored by performing blood and instrumental tests as per clinical practice.

### Mental assessment & psychometric instruments

All subjects were evaluated by one to two extensive diagnostic interviews at baseline and at W52. The diagnostic interviews systematically checked for the diagnostic criteria outlined by the DSM-IV of American Psychiatric Association (PA, USA) [19]. They were conducted by a senior specialist in psychosomatic medicine who was blinded to the patients’ dermatological status and psychometric results. The psychometric instruments used were the HADS for anxiety and depression [10]. The HADS utilizes a 14-item self-administered rating scale designed to identify and measure anxiety (seven items) and depression (seven items) separately, in physically ill individuals. Each item on the scale is scored from 0 to 3, so the total score for each subscale ranges between 0 and 21, and metal disease increases as the score rises. As reported by Zigmond *et al.*, a score of 7 or less is suggestive of a ‘non-case’, 8–10 a ‘doubtful case’ and 11 or more a definite ‘case’ or depression or anxiety [10].

### Statistical analysis

The target number of subjects was 30, with 15 subjects in the omalizumab group and 15 subjects in the control group, without any systemic treatment. The collected data are not distributed according to the normal curve (Shapiro–Wilk normality test: p < 0.05); for this reason, the statistical significance of the hypothesized comparisons was ascertained by means of the median test (YATES test) [19]. The differences were considered statistically significant for p-value <0.05. Statistical analyses were performed by SSPS V20 (Stat Corp, TX, USA).

## Results

**Figure 1.**
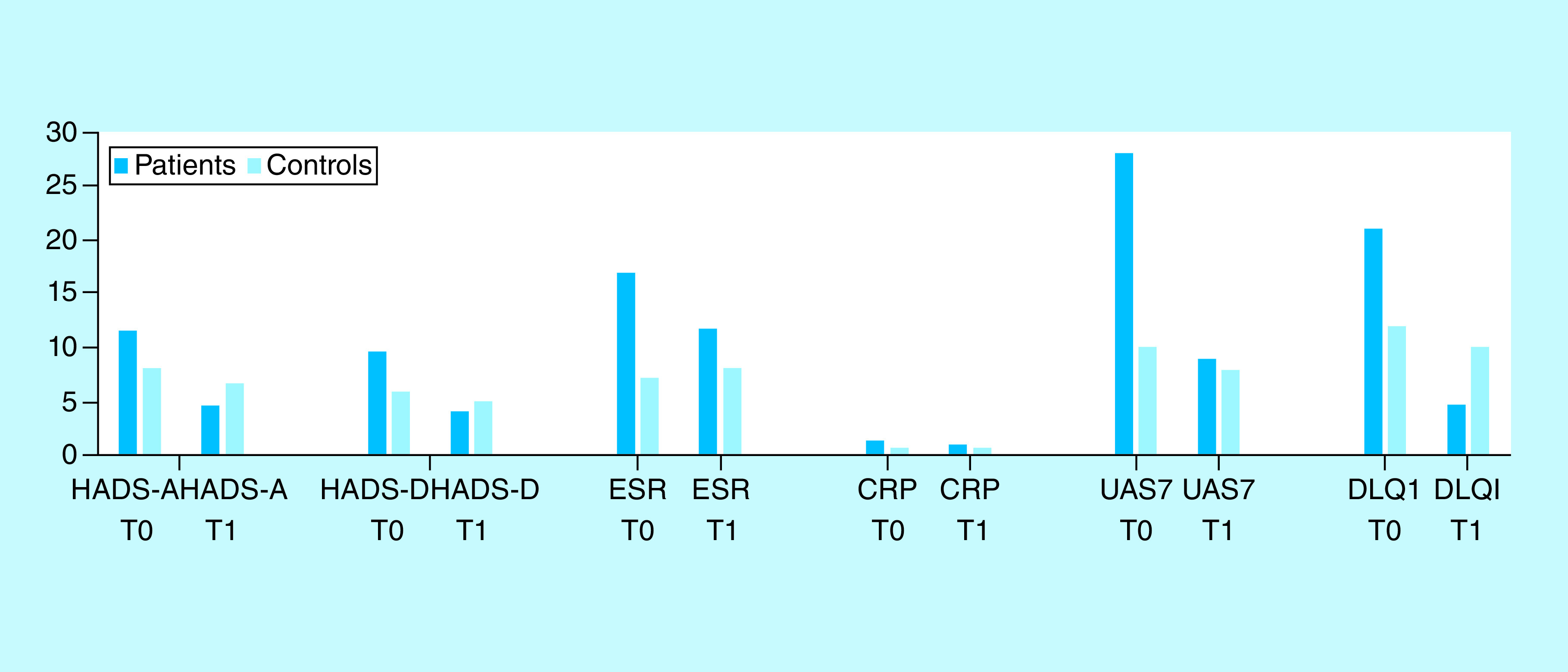
The case-control comparison of Hospital Anxiety and Depression Scale-Anxiety, Hospital Anxiety and Depression Scale-Depression, CRP, erythrocyte sedimentation rate, Urticaria Activity Score for 7 days and Dermatology Life Quality Index mean values at baseline (T0) and after 6 months of treatment with omalizumab (T1). DLQI: Dermatology Life Quality Index; ESR: Erythrocyte sedimentation rate; HADS: Hospital Anxiety and Depression Scale; HADS-A: HADS-anxiety; HADS-D: HADS-depression; UAS7: Urticaria Activity Score for 7 days.

Of the omalizumab group, ten subjects obtained an abnormal or borderline score for anxiety and seven individuals, an abnormal or borderline score for depression at baseline (T0). After 6 months of omalizumab (T1), six of ten (60%) subjects with a previous abnormal anxiety score, had a normal score for anxiety and five of seven (71%) subjects, who had obtained a borderline or abnormal depression score at T0, recorded a normal score. Mean values of the HADS at baseline were 11.66 for the anxiety subscale and 9.58 for the depression subscale. Mean values at T1 were 4.5 and 4, respectively, indicating a significant clinical improvement for the anxiety subscale (p < 0.01) and not a significant reduction for the depression symptoms (p > 0.05). Notably, we observed a pronounced decrease in the mean value of UAS7 from T0 (28) to T1 (8.75), mirroring a very significant improvement in subjective symptoms reported (p < 0.001). A similar trend can be observed for the DLQI score, with a mean value decreasing from 20.91 at T0 to 4.66 at T1 (p < 0.01). The ESR mean value significantly decreased as well from 16.91 mm/h at T0 in the group treated with omalizumab to 11.75 mm/h at T1 mean value (p < 0.01; ref see patients and method; normal range: 0–15 mm/h [male patients] or 0–20 mm/h [female patients]). Conversely, the CRP mean value decreased from 1.15 mg/l (T0) to 0.99 mg/l (T1), with a not significant reduction (p > 0.05; normal range: 0–8 mg/l). Therefore, this study did not demonstrate any acceptable statistical significance for these last two factors. No significant differences were observed in the control group before and after 6 months regarding the parameters of mental disorders and inflammation indexes (see Figure 1).

## Discussion

CSU patients demonstrated markedly reduced physical health and psychological health subscale scores compared with controls, confirming that such patients endure pronounced QoL impairment. QoL impairment in CSU patients was determined to be similar to acne patients and higher than in patients suffering from vitiligo [20]. In a prospective study of adult patients with CSU, psoriasis and atopic dermatitis, the physical discomfort associated with CSU was greater than that of psoriasis. In addition, daily living restrictions were greater in CSU than psoriasis [3]. Moreover, the percentage of dissatisfaction with their health state and with the treatment, was also higher in CSU patients than that of atopic dermatitis and psoriasis [21]. In our study, we observed higher levels of anxiety and depression in patients with CSU when compared with controls. These findings are consistent with those of previous studies conducted on patients with chronic urticaria [22]. Most reported cases experienced a stressor event within the months before the onset of the cutaneous manifestation [23]. Our findings align with data from the literature, as in our patient cohort, anxiety and depressive symptoms were secondary to stressful life events and triggered the emergence of urticarial symptoms in susceptible subjects. At baseline, in the omalizumab group, ten subjects reported an abnormal or borderline score for anxiety (HADS-A ≥8) and seven individuals an abnormal or borderline score for depression (HADS-D ≥8). After 6 months of treatment with omalizumab, 60% of subjects with a previous abnormal anxiety score achieved a normal score for anxiety and 71% of subjects who had obtained a borderline or abnormal score at T0 for depression obtained a normal score. In the omalizumab group, there was also a very significant reduction in the levels of UAS7 (p < 0.001) and a significant reduction in the Anxiety Scale, ESR and DLQI (p < 0.01) after therapy, but no significant decrease in the depression scale and CRP (p > 0.05). No significant differences were found in the control group before and after 6 months regarding the parameters of mental disorders and inflammation indexes. Some authors have previously identified that psychiatric comorbidity is a relevant driver of QoL impairment in patients with CSU. The reports by Engin *et al.* demonstrated that the severity of depression and anxiety, assessed by psychometric instruments, are positively correlated with the extent of QoL decrease in patients with CSU, suggesting that psychiatric comorbidity could influence mental health outcomes [7,8]. Our study clearly highlights that emotional distress is more pronounced and more commonly increased in patients with CSU than in the control group. Moreover, the participants’ statistic and continuous anxiety scores decreased significantly after treatment with omalizumab. Treating CSU patients with omalizumab allowed an improvement in UAS7 as well as in DLQI score.

As reported by several studies, in CSU patients there is a high prevalence of anxiety symptoms, due to the difficulties in coping with the disorder, impact of disease and therapy in their life, problems in interpersonal relationships, problems with emotional expression, occurrence of psychiatric disorders personality traits. Our CSU cohort was young and active, so the urticarial symptoms could afflict their professional life and emotional well-being [24]. After omalizumab therapy, we observed a meaningful decrease of Anxiety Scale and the patients reported feeling better and enjoying life more than before.

In our series, urticaria course parallels the one of depression, and response to treatment is inversely related to depression severity (data not shown). In patients affected by asthma, the treatment with omalizumab was correlated to depression resolution [25]. However, it should be noted that there is no evidence of a pharmacological effect on mental disorders. Thus, it seems likely that improvement is related to the reduction of asthmatic/urticarial symptoms, leading to an improvement in the QoL and psychological status [25]. Zabolinejad *et al.* noted that there is a communication between the serotonergic neuroendocrine system and the immune system, which may be involved in the pathogenesis of CSU through alterations in the expression of SERT. Thus, they speculated that modulating the level of SERT expression, for example, via SSRIs – which have been reported to be efficacious in treating inflammatory disorders of the skin as well as rheumatic disorders – could be effective in the treatment of CSU patients. Moreover, among CSU patients, the rate and intensity of SERT expression were significantly associated with the clinical severity of urticaria [26]. Thorslund *et al.* showed there was a significant relationship between the severity of psoriasis and the expression of SERT. These results, to some extent, can justify the possibility of a regulating role for SERT in the pathophysiology of inflammatory dermatoses [27]. Therefore, omalizumab could improve depression also acting on SERT expression, even though there is no evidence yet on this aspect. Further studies investigating its role are required to better understand its action. Regarding the response to omalizumab, we observed a correlation between a later response to treatment and the presence of depression. However, depressed patients normally minimize treatment results in terms of urticaria improvement, even though UAS7 decreases. We think this may be related to loss of confidence and interest in therapies, typical of a depressed state. For this reason, it is important to use validated tools, such as UAS7 to evaluate the response to treatment. ESR and CRP may represent further valid and objective biomarkers of the response to treatment. Regarding inflammatory biomarkers, such as ESR and CRP, we found a decrease of both, even if just ESR was significant, suggesting that omalizumab decreases urticaria signs and symptoms not just subjectively (as tested by UAS7), but also objectively. The limitations of our study are size of the cohort, lack of placebo group and its single-center nature. On the other hand, this study specifically addressed depression and anxiety in CU and our cohort was heterogeneous.

## Conclusion

Our study highlights the importance of investigating and managing mental comorbidities in the treatment of CSU, as this may result in a significant improvement of both psychiatric and dermatological symptoms. To this end, we would suggest a holistic approach to CSU patients, combining omalizumab with specific treatments for mental disorders. Further studies are needed and recommended to validate our suggestions.

## Future perspective

In our study, we demonstrated omalizumab efficacy in reducing both CSU and depression suggesting a new approach of combined therapy for these diseases. Indeed, CSU and depression are frequently associated and the existence of a unique therapy which ameliorates both the disorders could be interesting, not just for the therapeutic improvement, but also for the pathogenesis mechanisms involvement. Omalizumab could impact SERT expression and consequently the appearance of related mental disorders. Future research is needed to better evaluate the underlying pathways in common between CSU and depression. The recognition of a common mediator or pathway could be important for the prevention of both the diseases and for the development of new treatment strategies. In conclusion, we think our results if applied to a long-term study could lay the foundations for a new approach of combined therapy for CSU and mental disorders.

**Executive summary**

- Chronic spontaneous urticaria (CSU) and depression are frequently associated.
- Omalizumab improves both CSU and depression.
- Omalizumab as a new combined therapy for CSU and depression.
